# Comparing geospatial interpolation techniques for estimating ambient PM2.5 mass concentrations from sugarcane burning in South Florida

**DOI:** 10.21203/rs.3.rs-10743669/v1

**Published:** 2026-10-01

**Authors:** Anne Mielnik, Olivia Sablan, Jeffrey R. Pierce, Lilah M. Besser, Juyoung Park, Diana Mitsova, Diane Cook, Sherry WeMott, Christine L. Williams, Lisa A.K. Wiese, Sheryl Magzamen

**Affiliations:** Colorado State University; Colorado State University; Colorado State University; University of Miami School of Medicine; University of Arizona; Florida Atlantic University; Washington State University; Colorado State University; Florida Atlantic University; Florida Atlantic University; Colorado State University

**Keywords:** Air Pollution, Particulate Matter, Exposure Assessment, Kriging, Inverse Distance Weighting

## Abstract

Few studies quantify air pollutant exposures from agricultural burning – a potentially significant source of fine particulate matter (PM_2.5_) in rural regions – due to sparse air quality monitoring outside of urban areas. Using a dense network of low-cost sensors, we evaluated several interpolation-based approaches (e.g., inverse distance weighting and kriging) to estimate 24-hour average PM_2.5_ exposures from sugarcane burning in the Everglades Agricultural Area (EAA) for future regional health studies. Measurements were taken at 46 unique locations from June 8th, 2024, to December 1st, 2025, capturing the EAA’s burn and non-burn seasons. Model performance was evaluated using leave-one-out cross-validation and overall, varied by model and fit, meteorological season, and the presence of smoke, with R^2^ values ranging from 0 to 0.99. Based on the placement of sensors and number of measurements each day, inverse distance weighting models performed best – maintaining the highest R^2^ and the lowest root mean squared error and mean absolute error values for the most days. However, combining approaches, choosing the “best” model each day, provided the most robust exposure estimates for future studies. Independent of interpolation technique, estimated PM_2.5_ mass concentrations were highest in the presence of sugarcane burning smoke, increasing 1.65 μg/m^3^ (standard error = 0.13 μg/m^3^), and were localized inland, near sugarcane burning regions and the EAA. Notably, PM_2.5_ was lowest along the coast – highlighting air quality disparities in South Florida.

## Introduction

Florida is the largest producer of sugarcane in the United States, producing an average of 2.06 million tons of sugar per year, followed by Louisiana (1.78 million tons per year) and Texas (143,000 tons per year) (U.S. Department of Agriculture, 2025). Approximately 75% of Florida’s sugarcane is grown in Palm Beach County, with the remainder in Hendry, Glades, and Martin Counties – all of which include or are adjacent to the Everglades Agricultural Area (EAA) (Baucum et al., 2009). To ease sugar manufacturing – reducing silage before harvest, minimizing biomass for transport, and streamlining the milling process – over 400,000 acres of sugarcane in the EAA are burned each year (Baucum et al., 2009). As a result, smoke from prescribed burns for sugarcane crop management is a significant source of ambient fine particulate matter (i.e., particles with a diameter of 2.5 microns or less or “PM_2.5_”) in South Florida (Sablan et al., 2026; Slater et al., 2026; Nowell et al., 2022). Previous studies suggest air pollution from agricultural burning is associated with adverse health outcomes, including asthma, acute respiratory illness, and dementia (Boopathy et al., 2002; Jacobs et al., 1997; Mnatzaganian et al., 2015; Zhang et al., 2023). In South Florida, smoke exposure from sugarcane burning is also associated with mortality and notably, has higher smoke PM_2.5_ concentrations in low-income, rural areas (Nowell et al., 2022; Slater et al., 2026).

Despite this growing body of evidence, location-specific (e.g., residential or occupational) PM_2.5_ exposures from sugarcane burning have not been quantified to estimate personal exposures, which are needed to conduct individual-level health studies. For instance, although Nowell et al. (2022) conducted a health impacted assessment and estimated that PM_2.5_ from sugarcane fires contributes to mortality across South Florida. Similarly, Slater et al. (2026) found associations between smoke days and census tract-level demographics (e.g., indictors of poverty) by census tract; however, this study was also conducted on the county- or population-level and did not link PM_2.5_ exposures to health outcomes.

Previous studies have successfully estimated ambient PM_2.5_ exposures at point locations or fine spatial resolutions for health purposes, implementing geostatistics (e.g., kriging, land use regression), spatiotemporal models, chemical transport models, and/or machine learning (Liao et al., 2006; Kaufman et al., 2012; Zhang et al., 2023; Picciotto et al., 2024; Di et al., 2019). However, most epidemiological studies examine long-term PM_2.5_ exposures, failing to capture the effects of short-term or episodic events (e.g., agricultural burning) (Sangkham et al., 2024). In addition, many PM_2.5_ exposure models were built for the contiguous U.S. or for urban areas, and very few contain source-specific information, excluding those based on land use and/or traffic (Kaufman et al., 2012; Zhang et al., 2023; Di et al., 2019; Pawlowski et al., 2026; Esie et al., 2022; Di et al., 2016).

To elaborate, limited air quality research has been conducted in rural or agricultural areas due to lack of regulatory monitors (Roque et al., 2025). In epidemiological studies, exposure-response models necessitate estimates that support both time-resolved analyses for subclinical outcomes that lack recognizable symptoms and long-term integration for clinical outcomes such as diagnosis, health care utilization, and mortality. As a result, exposures are typically derived from geospatial interpolation of ground-based measures, with measurements collected daily and aggregated over longer, more specific periods (e.g., seasonally or annually) (O’Dell et al., 2024). As a result, challenges arise when ground-based data is sparse and monitors are set up to capture pollutants with predictable spatial and temporal patterns, such as traffic-related air pollutants. Conversely, sugarcane burning produces short-lived smoke plumes with high PM_2.5_ concentrations persisting for several hours before dissipating. Thus, evaluating approaches capable of capturing these frequent yet episodic, elevated PM_2.5_ exposures to support epidemiological studies is needed.

In this study, we estimated PM_2.5_ exposures from sugarcane burning in South Florida, providing short- and long-term exposure measures for future epidemiological studies. We leveraged ground-based PM_2.5_ mass measurements, satellite products, and public records to identify “smoke-impacted” days and to quantify location-specific PM_2.5_ concentrations attributable to sugarcane burning in the EAA. Further, we evaluated geospatial interpolation techniques for estimating 24-hour average PM_2.5_ mass concentrations, comparing the performance of inverse distance weighting (IDW), ordinary kriging (OK), and universal kriging (UK) models. Lastly, we explored combining approaches (i.e., choosing the “best” model for each day) and compared overall, combined model performance to individual model performance and to previous studies. To our knowledge, this is the first study to estimate location-specific PM_2.5_ exposures from agricultural burning at unmonitored locations in South Florida.

## Materials and Methods

### Ground-based PM _2.5_ measurements.

We deployed 44 PurpleAir sensors in Glades, Hendry, Okeechobee, and Palm Beach Counties to measure ambient PM_2.5_ mass concentrations from June 8th, 2024, to December 1st, 2025. Preliminary deployment of PurpleAir sensors and data quality procedures are described elsewhere (Sablan et al., 2026). Briefly, we downloaded 10-minute average humidity, temperature, and raw PM_2.5_ mass concentration data from online sensors using the PurpleAir Data Download Tool. Humidity (%) and temperature (°F) were calculated as the average measurement between channel A and channel B at each timestamp per sensor; whereas raw PM_2.5_ mass concentrations (μg/m^3^) were obtained for channels A and B separately. For offline sensors, 2-minute average data were uploaded from PurpleAir microSD cards at the end of deployment (throughout October 2025). Using both online and offline datasets, hourly and 24-hour average humidity, temperature, and raw PM_2.5_ mass concentrations were calculated for each sensor. Measurements were excluded from analysis if respective sensors failed to measure humidity and/or temperature. Further, individual data points were removed if they contained physically implausible values (e.g., relative humidity exceeded 100%, temperature was greater than 150°F or below − 50°F, etc.)

Additionally, we applied the Barkjohn correction factor to all quality-controlled raw PM_2.5_ mass concentration data (Barkjohn et al., 2021). Briefly, the Barkjohn correction factor calibrates PurpleAir sensor to EPA PM_2.5_ mass measurements, largely based on environmental conditions (e.g., relative humidity), with one calibration site located slightly northwest of the EAA (Barkjohn et al., 2021). Concurrent hourly and 24-hour average PM_2.5_ mass concentrations measured by two U.S. EPA Air Quality System (AQS) federal reference method (FRM) monitors within the study area were used to supplement PurpleAir data (U.S. Environmental Protection Agency, 2026). Locations of PurpleAir sensors and EPA AQS FRM monitoring sites are shown in Fig. 1.

### Open Burn Authorization records.

We requested Open Burn Authorization (OBA) records from the Florida Department of Agriculture and Consumer Services (FDACS) to investigate the timing and extent of agricultural burning from sugarcane crop management in Glades, Hendry, Highlands, Martin, Okeechobee, Palm Beach, and St. Lucie Counties from June 1st, 2024, to December 1st, 2025. OBAs are administered by the Florida Forest Service (FFS) for agricultural burning, silvicultural burning, land clearing, pile burning, and acreage burning; and specify the date, burn type, requested area, and location of each fire set. The date and time recorded on OBAs indicate when requests were authorized and typically corresponded to the date of burning; however, to accommodate wind and weather restrictions, some burns may be set a day or two afterwards (Florida Forest Service, 2023). Additionally, according to Florida state law, certified pile burns must be completed no later than one hour after sunset (e.g., between 5:30 PM EST and 8:30 PM EST, depending on season) or at least one hour before sunset if conducted in an area designated as “smoke sensitive” (i.e., if a hospital, school in session, or airport is within one-half mile of a proposed burn) (Wade & Mobley, 2007).

### Remote sensing observations.

We leveraged National Oceanic and Atmospheric Administration (NOAA) Hazard Mapping System (HMS) Fire and Smoke Product data to assess the timing and extent of prescribed-fire smoke across the study area (NOAA, 2025). As described by Sablan et al. (2026), HMS smoke plume polygons were used to determine the presence of smoke. NOAA HMS smoke data was downloaded from June 1st, 2024 to December 1st, 2025 (NOAA, 2025). Despite known limitations of this approach, including lack of vertical altitude data, interferences with cloud cover, and daytime-only observations, we found 197 days (36.4%) during the PM_2.5_ measurement period contained smoke plumes overlapping PurpleAir sensors or EPA AQS FRM monitors located in the study area.

### Identification of smoke-impacted days.

We evaluated the presence of smoke from sugarcane burning on a daily basis using multiple methods. Based on criteria established in Sablan et al. (2026), we designated the presence of smoke following:

1. 1. 15 μg/m^3^: A day at a specific monitor (and for the respective estimated PM_2.5_ surface) is designated as smoke-impacted when PM_2.5_ mass concentrations ≥ 15 μg/m^3^ for at least one hour within that 24-hour period.
2. 2. 20 μg/m^3^: A day at a specific monitor (and for the respective estimated PM_2.5_ surface) is designated as smoke-impacted when PM_2.5_ mass concentrations ≥ 20 μg/m^3^ for at least one hour within that 24-hour period.
3. 3. 30 μg/m^3^: A day at a specific monitor (and for the respective estimated PM_2.5_ surface) is designated as smoke-impacted when PM_2.5_ mass concentrations ≥ 30 μg/m^3^ for at least one hour within that 24-hour period.
4. 4. 20 μg/m^3^ or HMS: A day at a specific monitor (and for the respective estimated PM_2.5_ surface) is designated as smoke-impacted when either PM_2.5_ mass concentrations ≥ 20 μg/m^3^ for at least one hour within that 24-hour period or at least one NOAA HMS smoke plume polygon overlaps the monitor (**Figure S1**).

Due to the decreased number of days specific PurpleAir sensors and EPA AQS FRM monitors meet PM-based criteria (e.g., n_1_ = 63 days [11.6%], n_2_ = 24 [4.4%] days, and n_3_ = 7 [1.2%] days) compared to HMS-based criteria (197 days [36.4%]), criteria 4 (i.e., *20 μg/m*^*3*^ *or HMS*) was implemented to distinguish smoke-impacted days in statistical analyses (denoted “C1” in tables; n = 164 [30.3%] to 188 [34.7%] days based on model fit). Further, under more inclusive conditions (denoted “C2” in tables), a day was considered smoke-impacted if FFS administered an OBA for sugarcane crop management within the EAA administrative boundary defined by the South Florida Water Management District (South Florida Water Management District, 2018). During our study, OBAs were administered in the EAA for sugarcane crop management on 272 days or 50.2% of the time.

### Spatial interpolation of PM _2.5_.

We fit IDW, OK, and UK models to log transformed, corrected 24-hour average PM_2.5_ mass concentration data over a 5 km grid of the study area (9,500 km^2^ total area) for the entire measurement period. We used the gstat R package for IDW, semivariogram fitting for OK and UK, model cross-validation, and estimation of 24-hour average PM_2.5_ concentrations (Pebesma, 2004). Common geospatial model functions (e.g., Bessel, exponential, Gaussian, linear, and nugget) were considered to obtain optimal 24-hour average semivariogram parameters. Prior to fitting models, outliers were detected and removed using the Interquartile Range (IQR) method. Leave-one-out cross-validation (LOOCV) was used to evaluate model performance and calculate R^2^, root mean squared error (RMSE) and mean absolute error (MAE) values. With this, each measurement site was excluded, and models were re-run using data from the remaining 45 sites.

### Statistical analysis.

To evaluate the impacts of sugarcane burning on estimated PM_2.5_ mass concentrations, we performed Wilcoxon signed-rank tests on averaged PM_2.5_ mass concentrations for each 5 km grid cell in the study area comparing smoke-impacted to non-impacted days (using criteria “C1” and “C2”) and each model/fit function, resulting in 22 total tests.

Additionally, we explored combining interpolation methods using a semiautomated approach that implements all models and fits, choosing the “best” day based on the number of PM_2.5_ measurements, semivariogram goodness of fit (for kriging models), LOOCV values, and feasibility of PM_2.5_ estimates. For each day with estimated, gridded PM_2.5_ surfaces, output generated using each model/fit was compared by LOOCV values to determine “best” model/fit according to (1) highest R^2^, (2) lowest RMSE and MAE, (3) feasibility of PM_2.5_ estimates (i.e., impractical estimates were omitted) and (4) variance, especially near “hotspots” or in areas where measured 24-hour PM_2.5_ values were elevated. Further, IDW models were chosen on days when models/fits had comparable LOOCV values and PM_2.5_ estimates but were informed by less than 20 measurements.

Lastly, residential addresses from 766 participants enrolled in a supplemental health study funded by the National Institute on Aging were used to estimate and evaluate 24-hour average PM_2.5_ exposures at un-measured locations throughout the measurement period – resulting in 50 unique 5 km gridded locations for exposure assessment. All statistical analyses were performed using R (version 4.5.1) (R Core Team, 2025).

All values are reported in μg/m^3^ except for *n* (the number of 24-hour average PM_2.5_ measurements taken by PurpleAir sensors and EPA AQS FRM monitors). “C1” and “C2” denote different criteria for identifying smoke-impacted days: “C1” indicates [PM_2.5_] ≥ 20 μg/m^3^ for at least one hour within a 24-hour period or a NOAA HMS smoke plume overlapped a PurpleAir sensor or EPA monitor; “C2” shows an OBA for sugarcane crop management was administered within the EAA. Note: For 406 of 3,048 (13.3%) 24-hour average PM_2.5_ measurements during the “C1” smoke-impact classification, [PM_2.5_] exceeds primary annual National Ambient Air Quality Standards (NAAQS; 9 μg/m^3^); for 35 (1.14%) measurements, [PM_2.5_] exceeds secondary annual NAAQS (15 μg/m^3^) and for 1 measurement (0.032%), [PM_2.5_] exceeds primary and secondary 24-hour NAAQS (35 μg/m^3^).

## Results and Discussion

### PM _2.5_ measurements.

From June 8th, 2024, to December 1st, 2025, we collected 12,814 24-hour average PM_2.5_ mass measurements from all 44 PurpleAir sensors and two EPA AQS FRM monitors within the study area. Our data collection success rate for PurpleAir sensors was modest (53.8%). Data collection success rate was calculated as the number of “successful” measures passing QA/QC protocols divided by the number of total attempted measures (i.e., the number of hours between midnight on June 8th, 2024, and 23:59:00 PM EST on December 1st, 2025) multiplied by 100%. On occasion, some PurpleAir sensors failed to record measurements online, likely due to power outages, insufficient internet connection, or other factors related to deployment in rural areas. Specifically, a few participants hosting PurpleAir sensors unenrolled from the study due to moving out of the area (n = 2), death (n = 1), or loss of interest (n = 2), decreasing the number of sensors collecting measurements. Further, < 2% of online data failed quality control procedures, mostly toward the end of deployment. To help overcome these challenges, PurpleAir data from offline sensors were uploaded from microSD cards, with a recovery rate of 5%. As a result, the median number of PurpleAir sensors and EPA AQS FRM monitors recording PM_2.5_ mass measurements each day was 24 (range: 2 to 34). Summary statistics for PM_2.5_ mass measurements are shown in Table 1.

### OBA records.

During this study, 16,468 OBAs were administered in select South Florida counties, with a requested burned area of 730,234 acres. A total of 12,933 OBAs (78.5%) were administered for agricultural burning and 7,485 OBAs (45.4%) were administered for sugarcane crop management. Overall, OBAs were administered most in November and December 2024 (n = 1,084 per month), followed by November 2025 (n = 1,028), January 2025 (n = 927), February and March 2025 (n = 867 per month), October 2024 (n = 518), April 2025 (n = 444), October 2025 (n = 373), May 2025 (n = 265), and June 2024 (n = 28), with no OBAs administered in July, August, and September 2024 or June, July, August, and September 2025. Additionally, a majority of OBAs for sugarcane crop management were administered in Palm Beach County (n = 4,811 [64.3%]), followed by Hendry (n = 1,474 [19.7%]), Glades (n = 885 [11.8%]), Martin (n = 247 [3.3%]), Highlands (n = 62 [0.8%]), Okeechobee (n = 6 [0.1%]), and St. Lucie (n = 0) Counties.

In the EAA, 5,362 OBAs (32.5%) were administered during the study period. A total of 5,347 OBAs (99.7%) were administered for agricultural burning and 5,246 OBAs (97.8%) were administered for sugarcane crop management. Like the rest of South Florida, OBAs were administered most in the EAA between October and May, with no OBAs administered in June, July, August, or September.

### Estimated PM _2.5_ during smoke-impacted vs. non-smoke-impacted days.

Overall, 11 different geospatial interpolation techniques were used to estimate 24-hour average PM_2.5_ mass concentrations from June 8th, 2024, to December 1st, 2025, capturing 527 of 541 measurement days (97.4%). Summary statistics for estimated, gridded (i.e., area-weighted) PM_2.5_ mass concentrations for each model and fit function by meteorological season and smoke-impacted vs. non-smoke-impacted days are shown in Table 2 (**Tables S1 – S10**). Notably, output from all 11 models and fits indicate PM_2.5_ mass concentrations are highest in Spring (i.e., March, April, and May) and on smoke-impacted days. For the entire study period, estimated 24-hour average PM_2.5_ mass concentrations ranged from 0 to 23.8 μg/m^3^, excluding output from OK and UK models with Bessel and Gaussian fits, which frequently predicted negative values or concentrations beyond what is feasible in ambient air (i.e., on the order of 10^38^ μg/m^3^).

All values are reported in μg/m^3^ except for *n* (the number of estimated, gridded 24-hour average PM_2.5_ surfaces generated during the measurement period [of 541 days total]). “C1” and “C2” denote different criteria for identifying smoke-impacted days: “C1” indicates [PM_2.5_] ≥ 20 μg/m^3^ for at least one hour within a 24-hour period or a NOAA HMS smoke plume overlapped a PurpleAir sensor or EPA monitor; “C2” shows an OBA for sugarcane crop management was administered within the EAA. Spatial resolution is 2 km per grid cell.

Additionally, we found evidence of significant spatial variation in PM_2.5_ mass concentrations on smoke-impacted days, with the lowest concentrations observed in the eastern (coastal) part of the study area and the highest values inland, mostly within the EAA and/or adjacent to Lake Okeechobee (Fig. 2; **Figures S2 – S5**), as reported previously (Nowell et al., 2022; Sablan et al., 2026). Although Easterly trade winds prevail in summer (e.g., May to October) with a seasonal shift between Westerlies and prevailing winds from the North the rest of the year, local winds (i.e., sea breezes) push air/smoke inland during the day, when agricultural burning is permitted. Importantly, higher percentages of low-income, non-White individuals reside within or nearby the EAA compared to the coastline; not only highlighting the need for ambient air monitoring in sugarcane burning regions, but also further revealing disparities in PM_2.5_ smoke exposure among different ethnic, racial, and socioeconomic groups in South Florida.

Further, despite model/fit and criteria used to evaluate smoke-impacted days, Wilcoxon signed-rank tests indicate that for each grid cell in the study area, estimated average PM_2.5_ mass concentrations are higher on smoke-impacted days vs. non-smoke-impacted days (p < 0.001), suggesting sugarcane burning in the EAA is a statistically significant source of PM_2.5_ across the EAA. For all models/fits, estimated average PM_2.5_ mass concentrations range from 4.74 to 6.06 μg/m^3^ on smoke-impacted days vs. 3.17 to 4.33 μg/m^3^ on non-smoke-impacted days, increasing an average of 1.65 μg/m^3^ (standard error = 0.13 μg/m^3^), in the presence of prescribed-fire smoke. In previous studies, slight increases in PM_2.5_ were associated with adverse health effects, including neurological hospitalizations, perinatal outcomes, and decreases in workplace productivity (Shi et al., 2020; Gao et al., 2026; Hill et al., 2024).

Model performance varies by model/fit and season. Variograms for OK and UK models are shown in **Figures S6 and S7**. **Figure S8** shows scatterplots of measured vs. predicted PM_2.5_ mass concentrations at all PurpleAir and EPA AQS FRM monitoring sites during the study. Outliers are prevalent in OK and UK models with Bessel and Gaussian fits, oftentimes predicting values below 0 μg/m^3^ or on the order of 10^38^ μg/m^3^, which is unrealistic and out of agreement with other models. Additionally, OK models with a nugget fit predict the same value for every measurement each day, rendering this method unreliable for spatially varying data. Otherwise, internal coverage is generally good and appears consistent with LOOCV R^2^ values.

Summary statistics for LOOCV values from all 11 models are shown in Table 3. Overall, LOOCV R^2^ values are modest for all models/fits, ranging from 0 to 0.99. OK models with a nugget fit function report R^2^ values equal to 1, indicating a perfect fit, which is unlikely and misleading. Again, this is likely a result of models predicting the same value at every measured and un-measured location within the study area, which demonstrates poor generalizability for spatially varying data. The LOOCV R^2^ values vary by season for all other OK models, peaking in Fall (e.g., September, October, and November), and often surpass R^2^ = 0.50, and then decrease by 50% or more for the rest of the year (**Figure S9**). In terms of LOOCV R^2^, model performance is more consistent for IDW and UK models, increasing during months with smoke-impacted days (e.g., November to March) (**Figure S10**). LOOCV RMSE and MAE values are consistently low across models/fits (Table 3). For instance, using IDW, maximum RMSE and MAE are 3.33 μg/m^3^ and 3.21 μg/m^3^, respectively; using OK and UK models (with an exponential fit), maximum RMSE is 2.49 μg/m^3^ for both models and maximum MAE values are 1.30 μg/m^3^ and 1.29 μg/m^3^, respectively.

LOOCV values found in this study are comparable to or consistently lower than those reported elsewhere. To elaborate, Beelen et al. (2009) used OK, UK, and regression mapping to estimate PM_10_ mass concentrations across the European Union (EU; 2,854,116 km^2^) and found UK performed best, with R^2^ = 0.49 and RMSE = 5.19 μg/m^3^. Similarly, Kassteele et al. (2006) used UK to estimate PM_10_ mass concentrations over Western Europe, using a combination of groundbased measurements and satellite products, finding RMSE of 3.62 μg/m^3^ to 5.14 μg/m^3^ using different modeling procedures. In comparison, our UK models typically demonstrate better performance; however, we acknowledge comparing PM_2.5_ to PM_10_ over regional vs. continental scales is unrepresentative, given PM_10_ mass concentrations have more spatial and temporal variability than PM_2.5_, as PM_10_ is larger, has shorter atmospheric lifetimes (i.e., does not travel as far), and is emitted from local sources (e.g., traffic, dust, sea spray) (Pérez et al., 2016; Salvador, 2004). Additionally, the EU is ~ 2 million km^2^ larger than South Florida, and thus, any geostatistical approach is prone to yield higher error over larger areas when burdened with a similar ratio of land mass to air monitors.

In the U.S., several studies leveraging ground-based PM_2.5_ measures combined with statistical models have revealed disparities in air quality and monitoring in low-income, non-White communities within urban areas (Pawlowski et al., 2026; Esie et al., 2022). Specifically, Pawlowski et al. (2026) used GIS-based Kernel Density Estimation and spatial interpolation (i.e., IDW, spline, and kriging) to estimate PM_2.5_ mass concentrations in un-measured locations from EPA AQS monitoring data across the Greater Chicago Metropolitan area, noting limitations on IDW and kriging models based on the placement and number of monitors, as observed in our study. In the same region, Esie et al. (2022) deployed their own network of low-cost monitors to estimate PM_2.5_ mass concentrations using IDW, reporting similar results and limitations to Pawlowski et al. (2026), with the added drawback of missing data, which was also seen here. Unfortunately, neither study reported metrics on model performance. In the northeastern U.S., Di et al., (2016) used a hybrid chemical transport model and land use regression informed by EPA AQS monitoring data to estimate annual PM_2.5_ mass and elemental composition concentrations from 2001 to 2010, reporting R^2^ values ranging from 0.80 to 0.88 for PM_2.5_ mass. In general, PM_2.5_ concentrations were highest in urban areas and near major roadways, specifically the I-95 corridor, and in some coastal regions (i.e., Cape Cod and Long Island), and were lowest in rural areas (e.g., Maine, northern New Hampshire and Vermont) (Di et al., 2016).

Similar approaches have been implemented to estimate PM_2.5_ exposures from fire smoke. Across the contiguous U.S., Childs et al. (2022) developed machine learning models to estimate daily PM_2.5_ exposures from wildfire smoke for 2006 to 2020, observing increases in smoke PM_2.5_ by 5 μg/m^3^ in the western U.S., reporting model performance of R^2^ = 0.67 (overall) and R^2^ = 0.70 (using PurpleAir sensors only). Also, O’Dell *et al*. (2019) estimated seasonal mean PM_2.5_ from wildfire smoke using several approaches, observing between 1.2 μg/m^3^ and 9.2 μg/m^3^ of summer-mean PM_2.5_ from fires in the Pacific Northwest, depending on approach and year. In California, Picciotto et al. (2024) used chemical transport modelling (i.e., Community Multiscale Air Quality Modeling System [CMAQ]) to estimate 24-hour average PM_2.5_ exposures from wildland fire smoke at the ZIP code-level, finding increased exposures near forested regions and burned areas (e.g., in northern California and near Los Angeles), which are comparable to results observed here for sugarcane burning regions. Using models from the same study, 24-hour average wildland fire smoke PM_2.5_ concentrations from 2007 to 2013 were, on average, 1.22 μg/m^3^ (SD: 3.78 μg/m^3^), and accounted for ~ 25% of all-source-PM_2.5_ mass concentrations in California, varying by county (e.g., 4% in Orange County, near the affluent coastline, to 70% in Trinity County, northwest in the mountains) (Koman et al., 2019; Pawlowski et al., 2026). Further, other studies that research wildland fire smoke in California rely on nationwide ensemble-based models, which report an average 10-fold CV R^2^ value for annual PM_2.5_ mass estimates of 0.89 (Di et al., 2019). Lastly, in China, Yin et al. (2017) also found PM_2.5_ mass concentrations from crop residue burning correspond to seasonal burn patterns and are localized to burning regions. Thus, despite model complexity and study location, our approach and findings remain comparable to, if not as robust as and computationally less complex than previous studies.

Nonetheless, despite reasonable performance, errors in our exposure models will likely propagate to effect estimates in future epidemiological studies. Specifically, the tendency of OK and UK models with Bessel and Gaussian fits to overpredict PM_2.5_ concentrations on smoke-impacted days near “hotspots” or the EAA (with increased variance or error) would positively bias exposure-response models, overestimating health effects associated with sugarcane burning. Also, because PM_2.5_ exposure from sugarcane burning in the EAA varies significantly on an hourly basis, as observed by Sablan et al. (2026), our assignment of a single, 24-hour average exposure likely does not capture temporal variation in PM_2.5_ mass concentrations, resulting in time-activity error and thereby underestimating health effects associated with sugarcane burning.

All values are reported in μg/m^3^ except for *n* (the number of estimated, gridded 24-hour average PM_2.5_ surfaces generated) and *R*^*2*^.

Lastly, we explored combining methods using a semiautomated approach that implements all models/fits and chooses the “best” one each day based on the number of measurements, semivariogram goodness of fit (for kriging models), and finally, LOOCV values. For “combined” models, mean (SD) LOOCV values are: R^2^ = 0.93 (0.20), RMSE = 0.270 μg/m^3^ (0.278 μg/m^3^), and MAE = 0.210 μg/m^3^ (0.239 μg/m^3^). With this, 456 days (85.3% of measurement period) maintain R^2^ values above 0.50, which is modest model performance. Interestingly, for 450 days (84.2%), R^2^ > 0.70, and for 437 days (82.9%), R^2^ > 0.90, achieving excellent model performance. This approach results in steep improvements in model fit and performance compared to individual models and to previous studies, especially considering the episodic and spatially variable nature of sugarcane burning compared to other, more commonly modeled sources of PM_2.5_ (e.g., traffic, industrial emissions). Differences in model performance were primarily driven by days within non-burn or hurricane season, when PM_2.5_ concentrations lacked spatial heterogeneity across the study area and models were informed by a lower number of PM_2.5_ measurements. As a result, OK and UK models with a nugget fit were sometimes deemed “best,” despite model selection criteria, biasing model performance (towards R^2^ of 1) when model fit was potentially insufficient.

Consequently, when calculating PM_2.5_ exposures at 50 unique residential locations within the study area, IDW and most kriging models yield similar estimates on smoke-impacted and non-smoke-impacted days, with IDW resulting in larger differences during smoke-impact vs. non-smoke-impacted days. For example, using IDW, 24-hour average PM_2.5_ exposures range from 0 μg/m^3^ to 18.0 μg/m^3^, with mean (SD) of 5.52 μg/m^3^ (2.55 μg/m^3^) and 3.31 μg/m^3^ (2.17 μg/m^3^) on smoke-impacted and non-smoke-impacted days, respectively. In comparison, OK models with an exponential fit resulted in 24-hour average PM_2.5_ exposures ranging from 0.001μg/m^3^ to 16.2 μg/m^3^, with 1.21μg/m^3^ (0.023 μg/m^3^) and 1.20 μg/m^3^ (0.031 μg/m^3^) on smoke-impacted and non-smokeimpacted days, respectively; and OK models with a linear fit resulted in 24-hour average PM_2.5_ exposures ranging from 0 μg/m^3^ to 22.7 μg/m^3^, with 1.23 μg/m^3^ (0.089 μg/m^3^) and 1.21 μg/m^3^ (0.111 μg/m^3^) on smoke-impacted and non-smoke-impacted days, respectively. Further, UK models with an exponential fit resulted in 24-hour average PM_2.5_ exposures ranging from 0.001μg/m^3^ to 23.8 μg/m^3^, with 1.27 μg/m^3^ (0.117 μg/m^3^) and 1.24μg/m^3^ (0.138 μg/m^3^) on smoke-impacted and non-smoke-impacted days, respectively; and UK models with a linear fit resulted in 24-hour average PM_2.5_ exposures ranging from 0 μg/m^3^ to 23.5 μg/m^3^, with 1.28 μg/m^3^ (0.166 μg/m^3^) and 1.25 μg/m^3^ (0.181 μg/m^3^) on smoke-impacted and non-smoke-impacted days, respectively.

## Conclusions

Ultimately, IDW models consistently performed as well as or better than OK models or analogous UK models, on the basis of (1) the total number of days PM_2.5_ mass concentrations were estimated using this approach (n = 527 of 541 days [97.4%]) compared to OK and UK models (n = 436 days [80.5%] to 445 days [82.2%]); (2) estimated PM_2.5_ mass concentrations calculated by IDW were reasonable compared to other models, some of which predicted unrealistic values; and (3) IDW models yielded the highest number of “best” days (with the highest LOOCV R^2^ and lowest RMSE and MAE values compared to other models). However, we acknowledge these findings may be a result of IDW requiring fewer 24-hour average PM_2.5_ measurements (n_min_ = 5 in this study) than kriging (n_min_ = 20), which inherently excludes days with less data from kriging models and may artificially inflate LOOCV R^2^ values for IDW (e.g., R^2^ values calculated from three data points are typically high [> 0.9]). Overall, the best LOOCV R^2^ values for all models varied day-to-day and by meteorological season and were highest on smoke-impacted days using IDW and UK. This not only indicates low spatial heterogeneity in PM_2.5_ mass concentrations across the study area on non-smoke-impacted days, revealing a lack of “hotspots” or localized emission sources in certain months (e.g., summertime), but also highlights approaches based on spatial variation, such as IDW and kriging, underperform in certain locations/scenarios (e.g., rural areas with few or small emission sources).

LOOCV RMSE and MAE values were consistently low across models/fits yet comparable to those reported elsewhere (Beelen et al., 2009; Di et al., 2016, 2019; Kassteele et al., 2006; Liao et al., 2006; Mercer et al., 2011). However, we acknowledge comparisons to previous studies are limited based on differences in particle sizes investigated and study areas. Nonetheless, our study is still subject to similar exposure assessment errors on future epidemiological studies, as mentioned previously.

Despite these limitations, our study maintains several key strengths. First, our exposure models rely on direct PM_2.5_ mass measurements located in or near the EAA. This is a rare advantage, as most exposure assessment studies are conducted in urban areas, relying on sparsely distributed EPA AQS monitoring data and/or lack direct smoke measures near agricultural burns. Direct measurements of prescribed-fire smoke are novel and useful, as sugarcane burning in the EAA is a persistent source of PM_2.5_ emitted to an otherwise lowly polluted airshed, offering a unique opportunity to characterize and quantify these oftentimes uncaptured emissions. Further, an added strength of this framework is its generalizability to other sparsely monitored regions where episodic burning occurs. Combining calibrated low-cost PurpleAir measurements with satellite-derived fire and smoke plume detections addressed common challenges to rural and agricultural areas nationwide, including sparse air monitoring and satellite interferences, while remaining cost-effective for largescale health studies. Accordingly, this framework could be implemented in other sugarcane-producing regions, such as Louisiana and Texas, as well as agricultural burning regions across the Midwest and Great Plains. However, because this approach estimates 24-hour average PM_2.5_ exposures and does not account for high-resolution variables, including atmospheric transport or meteorology, it is best-suited for relatively flat landscapes and may require modification (e.g., averaged to hourly) in regions where terrain or wind strongly influence pollutant dispersion (e.g., mountain regions or deserts).

Second, our approach is semiautomated, implementing several geospatial interpolation techniques and fit functions for each day over the entire sampling period. This helps to overcome modest success rate in sample collection and successfully captures PM_2.5_ mass concentrations (R^2^ > 0.9) in un-measured locations over 80% of the time by leveraging a combination of IDW and kriging models. In addition, exposure models met our objective of quantifying 24-hour average, location-specific (i.e., residential) PM_2.5_ mass concentrations intended for individual-level health studies investigating short-term outcomes, specifically cognitive function in intermittent burn areas.

Finally, regardless of model/fit, performance, and criteria used to evaluate smoke-impacted days, Wilcoxon signed-rank tests indicate that for each grid cell in the study area, estimated mean PM_2.5_ mass concentrations are higher on smoke-impacted days vs. non-smoke-impacted days, suggesting sugarcane burning in the EAA is a statistically significant and meaningful source of PM_2.5_ in South Florida – further highlighting these methodologies and findings may be insightful for other parts of the Southeastern U.S. where sugarcane burning is prevalent.

## Supplementary Material

Supplementary Files

This is a list of supplementary files associated with this preprint. Click to download.

- SI.docx

## Figures and Tables

**Figure 1:**
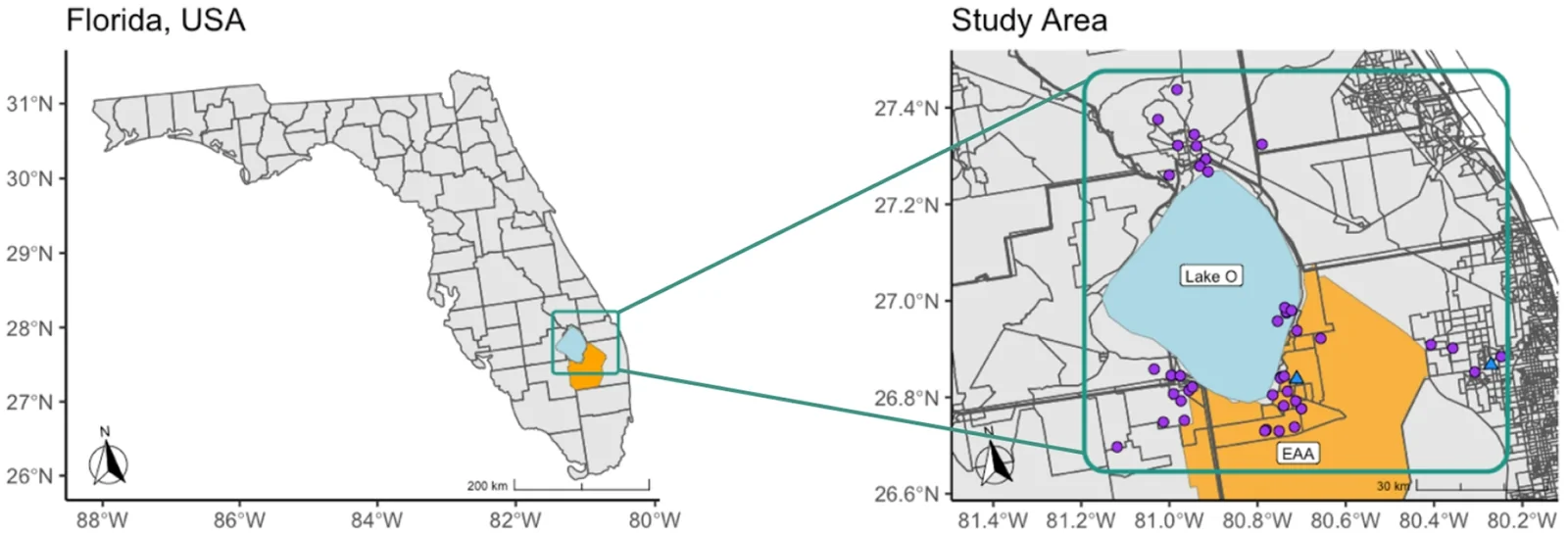
Map of Florida with county lines (left) and the study area (right) with county lines (dark gray, bold), census block groups (gray), PurpleAir sensors (purple circles) and EPA AQS FRM monitors (blue triangles). In both panels, the study area boundary, Lake Okeechobee (“Lake O”), and the Everglades Agricultural Area (“EAA”) are shown in mint, light blue, and orange, respectively. Locations of PurpleAir sensors were jittered by 5 km to protect participant privacy.

**Figure 2:**
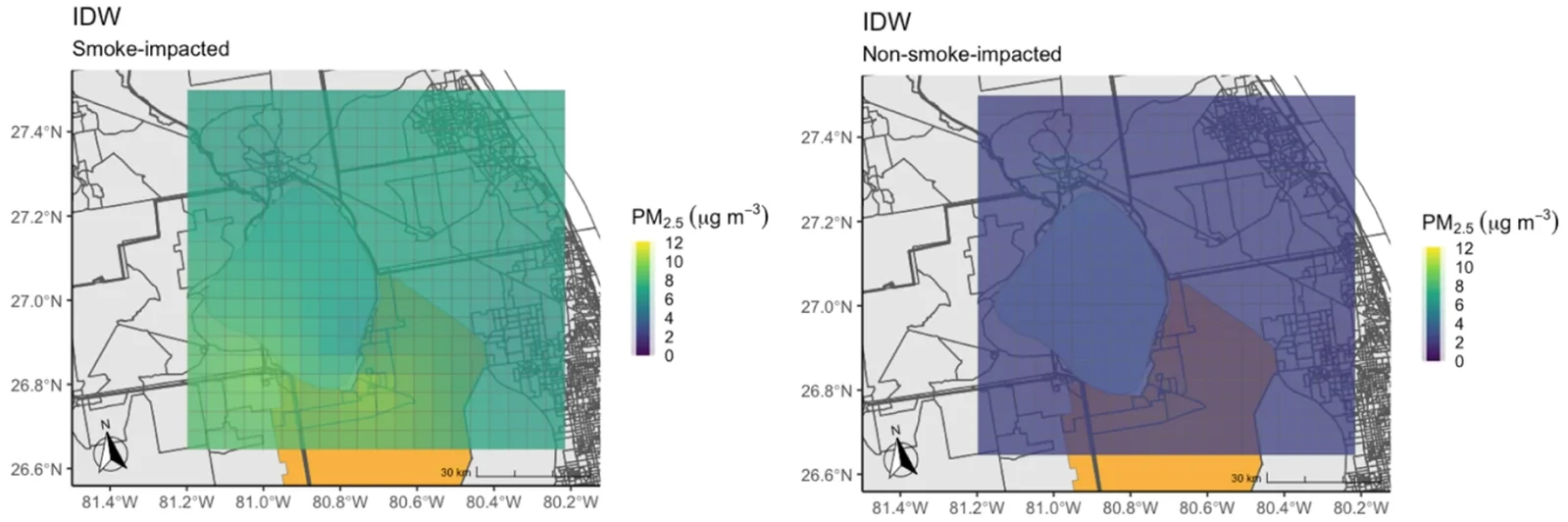
Estimated 24-hour average PM_2.5_ concentrations using IDW on smoke-impacted vs. non-smoke-impacted days. November 25^th^, 2025, represents the “smokeimpacted” designation and June 30^th^, 2024 represents the “non-smoke-impacted” designation under both “C1” and “C2” criteria.

**Table 1:** Summary statistics for 24-hour average PM_2.5_ measurements by meteorological season, by smoke-impacted vs. non-smoke-impacted days, and overall, for the entire study period.

	n	Mean	SD	Min.	Q1	Median	Q3	Max.
***Meteorological season***								
Spring (MAM)	2,852	5.94	2.69	0	4.11	5.50	7.48	23.3
Summer (JJA)	3,350	4.09	2.92	0	2.51	3.52	4.82	66.2
Fall (SON)	3,478	4.13	3.99	0	2.28	3.40	5.10	120.
Winter (DJF)	3,134	5.01	2.75	0	3.31	4.64	6.11	23.5
**Smoke-impacted vs. non-smoke-impacted days**								
Smoke (C1)	3,048	6.06	3.06	0	3.99	5.56	7.62	47.8
No smoke (C1)	9,766	4.33	3.20	0	2.60	3.82	5.37	120.
Smoke (C2)	7,750	5.38	3.46	0	3.41	4.80	6.75	120.
No smoke (C2)	5,264	3.82	2.67	0	2.28	3.40	4.70	66.2
**Overall**	**12,814**	**4.74**	**3.25**	**0**	**2.84**	**4.16**	**5.98**	**120.**

**Table 2:** Summary statistics for estimated 24-hour average PM_2.5_ concentrations from IDW models by meteorological season, by smoke-impacted vs. non-smoke-impacted days, and overall, for the entire study period.

Spring (MAM)	n	Mean	SD	Min.	Q1	Median	Q3	Max.
	92	5.75	2.07	1.20	4.22	5.21	6.94	15.1
Summer (JJA)	162	3.52	1.93	0	2.45	3.32	4.09	14.4
Fall (SON)	182	3.20	2.32	0	1.80	2.93	4.42	15.5
Winter (DJF)	91	4.58	2.59	0	3.31	4.37	5.31	20.6
Smoke (C1)	188	5.23	2.38	0	3.64	4.85	6.62	20.5
No smoke (C1)	339	3.28	2.11	0	2.01	3.20	4.26	17.6
Smoke (C2)	272	4.74	2.47	0	3.21	4.45	6.07	20.6
No smoke (C2)	255	3.17	2.04	0	1.94	3.03	4.05	14.4
**Overall**	**527**	**3.98**	**2.40**	**0**	**2.54**	**3.72**	**5.07**	**20.6**

**Table 3:** Summary statistics for LOOCV values from 11 different geospatial interpolation models representing the entire study period.

	n	Mean	SD	Min.	Q1	Median	Q3	Max.
*R* ^*2*^								
**IDW**	527	0.19	0.19	0	0.03	0.12	0.30	0.93
**OK**								
Bessel	445	0.20	0.27	0	0.01	0.09	0.25	0.99
Exponential	445	0.24	0.29	0	0.02	0.12	0.31	0.99
Gaussian	445	0.17	0.24	0	0.01	0.06	0.21	0.99
Linear	445	0.15	0.18	0	0.02	0.08	0.21	0.96
Nugget	436	1	0	1	1	1	1	1
**UK**								
Bessel	445	0.15	0.15	0	0.02	0.09	0.24	0.80
Exponential	445	0.16	0.16	0	0.03	0.11	0.25	0.79
Gaussian	445	0.14	0.16	0	0.01	0.07	0.22	0.81
Linear	445	0.16	0.16	0	0.02	0.09	0.25	0.81
Nugget	436	0.15	0.16	0	0.01	0.08	0.23	0.81
**RMSE (μg/m^3^)**								
**IDW**	527	0.295	0.292	0.011	0.156	0.215	0.307	3.33
**OK**								
Bessel	445	0.271	0.258	0.060	0.158	0.225	0.295	3.73
Exponential	445	0.261	0.207	0.059	0.155	0.218	0.289	2.49
Gaussian	445	0.308	0.342	0.060	0.164	0.235	0.338	4.33
Linear	445	0.261	0.202	0.062	0.156	0.222	0.289	2.46
Nugget	436	0.264	0.188	0.059	0.156	0.227	0.295	2.17
**UK**								
Bessel	445	0.276	0.269	0.006	0.157	0.218	0.305	3.67
Exponential	445	0.265	0.222	0.064	0.155	0.209	0.291	2.49
Gaussian	445	0.309	0.313	0.066	0.164	0.236	0.344	3.83
Linear	445	0.265	0.216	0.069	0.157	0.215	0.300	2.43
Nugget	436	0.266	0.209	0.059	0.158	0.219	0.300	2.19
**MAE (μg/m^3^)**								
**IDW**	527	0.229	0.247	0.008	0.120	0.164	0.233	3.21
**OK**								
Bessel	445	0.207	0.173	0.048	0.123	0.173	0.231	2.54
Exponential	445	0.198	0.136	0.047	0.120	0.166	0.224	1.29
Gaussian	445	0.230	0.220	0.048	0.127	0.183	0.249	2.76
Linear	445	0.199	0.134	0.050	0.121	0.170	0.228	1.34
Nugget	436	0.204	0.125	0.047	0.126	0.181	0.229	1.08
**UK**								
Bessel	445	0.209	0.180	0.051	0.120	0.161	0.230	2.42
Exponential	445	0.199	0.148	0.046	0.117	0.155	0.221	1.30
Gaussian	445	0.228	0.207	0.053	0.127	0.176	0.261	2.23
Linear	445	0.200	0.145	0.052	0.120	0.158	0.223	1.30
Nugget	436	0.203	0.145	0.048	0.122	0.165	0.231	1.17

## Data Availability

PurpleAir data will be made available upon request. EPA AQS monitoring data is publicly available at https://www.epa.gov/outdoor-air-quality-data. OBAs were directly requested from FDACS, who are reachable online at https://www.fdacs.gov/Contact-Us/Public-Records-Requests. NOAA HMS Fire and Smoke Product data is publicly available for download at https://www.ospo.noaa.gov/products/land/hms.html#data. Shape files for EAA administrative boundaries defined by the South Florida Water Management District can be found at https://geodata.floridagio.gov/datasets/sfwmd::everglades-agricultural-area-eaa-boundary/about.
